# Effects of Nutritional Conditions and Exogenous N-Acyl-Homoserine Lactones on the Resuscitation of Sodium Hypochlorite-Induced Dormant *Vibrio parahaemolyticus*

**DOI:** 10.3390/microorganisms14081779

**Published:** 2026-08-12

**Authors:** Shuangping Li, Jiantao Cao, Rundong Wang, Yijia Deng, Jinhua Ye, Chen Wang, Shumin Liu, Biaoshi Wang, Ahmed S. M. Saleh, Ravi Gooneratne, Xuepeng Li, Jianrong Li

**Affiliations:** 1 College of Food Science and Engineering, Lingnan Normal University, Zhanjiang 524048, China; 2 Jinan Center for Agricultural Product Quality and Safety, Jinan 250001, China; 3 College of Food Science and Engineering, Bohai University, Jinzhou 121013, China; 4 Department of Wine, Food and Molecular Biosciences, Faculty of Agriculture and Life Sciences, Lincoln University, Lincoln 7647, New Zealand

**Keywords:** *Vibrio parahaemolyticus*, viable but non-culturable, sodium hypochlorite, N-acyl-homoserine lactones, resuscitation, quorum sensing, aquatic products

## Abstract

This study investigated the effects of nutritional conditions and exogenous N-acyl-homoserine lactones (AHLs) on the recovery of sodium hypochlorite (NaClO)-induced viable but non-culturable (VBNC) *Vibrio parahaemolyticus.* Exposure to 40 mg/L NaClO for 20 min resulted in the loss of culturability while viable cells (approximately 1.19 × 10^2^ cells/mL) remained detectable by SYTO9/PI staining, consistent with the operational criteria for the VBNC state. Five resuscitation systems were evaluated, including tryptic soy broth (TSB) alone, heat treatment, TSB supplemented with sodium pyruvate, TSB supplemented with Tween 80, and TSB supplemented with five AHLs (C4-HSL, C6-HSL, C8-HSL, OHHL, and 3-oxo-C14-HSL). Among the tested systems, the AHL-supplemented medium showed the highest recovery efficiency, characterized by a shortened lag phase, colony characteristics and cellular morphology approaching those of normal cells, increased intracellular ATP levels, and respiratory chain activity approaching normal levels. RT-qPCR analysis showed significantly increased expression of quorum sensing- and stress response-related genes, including opaR, atpA, soxR, and katG, in the AHL-treated group compared with the TSB group. These findings suggest that AHL supplementation is associated with enhanced recovery of VBNC *V. parahaemolyticus* and may facilitate metabolic reactivation during this process.

## 1. Introduction

*Vibrio parahaemolyticus* (Vp) is recognized as a major foodborne pathogen that is widely distributed in marine organisms, including fish and shellfish. The consumption of contaminated raw or undercooked seafood is associated with acute gastroenteritis, and in severe cases, may result in septicemia or death, thereby posing a significant and ongoing threat to public health and seafood safety [1].

During food processing, transportation, and cold-chain storage, microorganisms are frequently exposed to multiple environmental stresses, including low temperature, nutrient limitation, osmotic pressure changes, and disinfectant residues. In response to such adverse conditions, some bacteria may transition into a dormant physiological state known as the viable but non-culturable (VBNC) state. Cells in this state are unable to form colonies on conventional culture media but maintain intact membrane integrity, basal metabolic activity, and the presence of virulence-associated genes [2]. When environmental conditions become favorable, VBNC cells can resuscitate and return to a culturable state, regaining their capacity for growth and proliferation [3]. The VBNC state is widely regarded as a bacterial survival strategy that enables pathogenic bacteria to persist under adverse environmental conditions, and its potential public health implications have been well documented [4,5]. This characteristic can result in false-negative outcomes in traditional plate-counting methods, thereby significantly increasing food safety risks. To date, a wide range of bacteria, including Salmonella, Staphylococcus aureus, and Escherichia coli, have been reported to enter the VBNC state [6,7,8].

Sodium hypochlorite (NaClO) is widely used for the disinfection of ready-to-eat seafood products, including salmon, oysters, and tiger prawns, as well as food processing equipment, due to its low cost and broad-spectrum bactericidal activity. However, oxidative stress induced by sublethal NaClO treatment has been reported to be associated with VBNC state formation in several aquatic pathogens, raising concerns about the efficacy of routine disinfection practices [9,10]. Zheng et al. [11] reported that treatment of *Salmonella enteritidis* with 40 mg/L available chlorine for 25 min induced the bacteria into a VBNC state, accompanied by morphological alterations characterized by wrinkling and collapse of the cell surface. In contrast, exposure to concentrations exceeding 60 mg/L resulted in complete cell death, with evident structural disruption and breakage. Furthermore, Lu et al. [12] found that VBNC-*S. enteritidis* could restore normal metabolic function under specific resuscitation conditions. These findings indicate that inappropriate use of disinfectant concentrations may induce foodborne pathogens into a VBNC state, thereby increasing the risk of subsequent resuscitation. However, whether NaClO can induce Vp to enter the VBNC state, as well as the conditions required for its resuscitation, remain largely unexplored.

Nutritional conditions are considered an important factor influencing the recovery of VBNC cells by providing energy substrates and other nutrients that may support metabolic reactivation [13,14]. Yan et al. [15] found that varying concentrations of tryptic soy broth (TSB) could resuscitate VBNC-*S. aureus*, with resuscitation efficiency increasing in a concentration-dependent manner. Zhang et al. [16] reported that sodium pyruvate, a reactive oxygen species (ROS) scavenger, significantly enhanced resuscitation efficiency, possibly by supplying energy substrates and mitigating oxidative damage. In addition, Zhao et al. [17] reported that a temperature upshift combined with supplementation of Tween 80 effectively restored VBNC *Listeria monocytogenes* cells. Collectively, these findings suggest that appropriate nutrient availability and specific chemical additives play important roles in facilitating the resuscitation of bacteria from the VBNC state.

Quorum sensing (QS) is a regulatory mechanism by which bacteria produce and detect autoinducers to coordinate gene expression in a cell-density-dependent manner. In Gram-negative bacteria, N-acyl-homoserine lactones (AHLs) are the core QS signaling molecules, regulating a wide range of physiological traits and metabolic activities [18]. Recent studies suggest that QS signaling may contribute to the transition of bacteria between dormant and active states, including the recovery of VBNC cells [19,20]. Previous studies [21,22] found that VBNC-Vp cells formed during raw salmon processing could resuscitate during storage, and that the concentrations of five AHLs (C4-HSL, C6-HSL, C8-HSL, OHHL, and 3-oxo-C14-HSL) were positively correlated with the resuscitation rate of dormant cells. These findings suggest that this group of five AHLs may play a role in triggering the resuscitation of VBNC-Vp. However, the specific effects and underlying mechanisms of these signaling molecules in VBNC resuscitation remain to be elucidated.

The regulator OpaR is a central component of the quorum sensing system in Vp. The opaR gene, which encodes the OpaR protein, directly regulates multiple physiological processes, including biofilm formation, virulence expression, and metabolic adaptation [23]. Previous studies have shown that opaR expression is modulated by environmental stress and is associated with changes in the physiological and metabolic state of Vp cells [24]. The energy-metabolism-related gene atpA encodes the α-subunit of ATP synthase, a key enzyme in ATP production, and its expression level is closely associated with intracellular ATP concentration and overall metabolic activity [25]. The oxidative stress regulator SoxR functions as a sensor of intracellular reactive oxygen species (ROS) and activates the transcription of downstream antioxidant genes, thereby contributing to cellular protection against oxidative damage [26]. In addition, the katG gene encodes catalase, which catalyzes the decomposition of H_2_O_2_, facilitates ROS scavenging, and supports the repair of oxidative stress-induced damage [27]. These genes have been reported to be associated with the physiological status of VBNC cells and their response to resuscitation stimuli, making them useful molecular indicators in VBNC studies [28,29].

In this study, NaClO-induced VBNC-Vp was used as a model system to investigate the effects of different nutritional conditions and the five AHLs described above on the resuscitation of dormant bacteria. Growth kinetics, colony morphology, cell ultrastructure, cell viability, intracellular ATP levels, respiratory chain activity, and the expression of key genes (opaR, atpA, soxR, and katG) were systematically assessed using RT-qPCR to comprehensively evaluate differences in resuscitation efficiency under varying nutrient conditions and signaling molecule treatments.

The aim of this study was to elucidate the potential mechanisms by which exogenous AHLs promote VBNC-Vp resuscitation at the phenotypic, metabolic, and molecular levels, and to identify an optimal culture system for enhancing the recovery of VBNC cells. The findings are expected to provide a theoretical basis for optimizing disinfection strategies in aquatic products and improving methodologies for the detection and resuscitation of dormant Vp.

## 2. Materials and Methods

### 2.1. Bacterial Strain and Culture Conditions

The *Vibrio parahaemolyticus* strain used in this study was Vp RS1 (GDMCC No. 1.3664), originally isolated from raw salmon and preserved in the Laboratory of Bulk Aquatic Product Storage and Preservation, Bohai University. Frozen stock cultures stored at −80 °C were thawed and streaked onto tryptic soy agar (TSA, Guangdong Huankai Biotechnology Co., Ltd. [GHB], Guangzhou, China) plates, followed by incubation at 37 °C for 24 h in an inverted position. A single colony was then inoculated into 20 mL of sterile tryptic soy broth (TSB, GHB, Guangzhou, China) and cultured at 37 °C with shaking at 160 r/min (ZQFW-70, Tianjin Laiboteri Instrument Co., Ltd., Tianjin, China) for 16 h. Subsequently, the culture was transferred to fresh TSB at an inoculum size of 1% (*v*/*v*) and incubated for an additional 4.5 h to reach the logarithmic growth phase, at which point the cell density was approximately 2 × 10^8^ CFU/mL.

### 2.2. Induction of VBNC State by Sodium Hypochlorite

The induction was performed according to the method of Arvaniti et al. [19] with minor modifications. Bacterial cultures at the logarithmic growth phase were aliquoted into sterile centrifuge tubes (15 mL per tube), centrifuged at 4 °C and 8000× *g* for 20 min (Avanti J-E, Beckman Coulter, Brea, CA, USA), and the supernatant was discarded. The cell pellets were washed twice with sterile phosphate-buffered saline (PBS) and resuspended to adjust the cell density to approximately 10^6^ CFU/mL. The NaClO stock solution (effective chlorine content ≥ 5%, Sinopharm Chemical Reagent Co., Ltd., Shanghai, China) was diluted with distilled water to final available chlorine concentrations of 10, 20, 30, 40, 50, 60, and 70 mg/L. The actual available chlorine concentration was verified using the DPD method. Equal volumes (15 mL) of each NaClO solution were added to the cell pellets, mixed thoroughly, and incubated at room temperature in the dark for 20 min. The reaction was terminated by the addition of 0.12% (*v*/*v*) sodium thiosulfate (Sinopharm Chemical Reagent Co., Ltd., Shanghai, China) to neutralize residual free chlorine. The control group was treated with sterile PBS instead of NaClO. Following treatment, the bacterial suspensions were washed twice with PBS and subsequently used for the determination of culturability and morphological observation.

### 2.3. Verification of the VBNC State

Culturable cell counts were determined using thiosulfate citrate bile salt sucrose (TCBS, GHB, Guangzhou, China) agar. NaClO-treated bacterial suspensions were serially diluted tenfold, and 100 μL aliquots were spread onto TCBS plates, followed by incubation at 37 °C for 24 h. In the absence of detectable colony formation, SYTO9/PI dual fluorescence staining (SYTO9/PI dual-staining kit, Beyotime Biotechnology Co., Ltd., Shanghai, China) in combination with laser confocal scanning microscopy (TCS SP8, Leica, Wetzlar, Germany) was used to evaluate cell membrane integrity and indicate the induction of the VBNC state.

Cell viability was assessed using SYTO9/PI dual fluorescence staining, in which SYTO9 labels viable cells and propidium iodide (PI) labels membrane-compromised cells [30]. The VBNC state was identified according to the standard operational criteria based on the combined assessment of (i) loss of culturability on TCBS agar, (ii) retention of membrane integrity as determined by SYTO9/PI staining (≥90% green-fluorescent cells), and (iii) the presence of basal metabolic activity. This multi-parameter is widely used for the operational identification of VBNC cells in *Vibrio* species and other bacteria [31,32,33,34]. Briefly, 1 mL of bacterial suspension was centrifuged at 4 °C and 8000 r/min for 3 min (Avanti J-E, Beckman Coulter, Brea, CA, USA), the supernatant was discarded, and the cell pellet was washed twice with sterile PBS. The pellet was resuspended in 1 mL of sterile PBS, and SYTO9 and PI dyes were added to final concentrations of 5 μmol/L each. After incubation in the dark for 15 min, a 10 μL aliquot of the stained suspension was placed on a glass slide, covered with a coverslip, and observed using a laser confocal scanning microscope (TCS SP8, Leica, Wetzlar, Germany). Ten random fields of view were selected for image acquisition, and at least 50 bacteria were counted per field. Green and red fluorescent cells were counted separately. The VBNC state was verified when the proportion of green fluorescent cells was ≥90% in the absence of detectable colonies on TCBS plates. The number of VBNC cells and the VBNC incidence rate were calculated using Equations (1) and (2) as follows:

a = b − c (1)

D (%) = a/b × 100 (2)

where a represents the number of VBNC cells (CFU/mL), b denotes the number of viable cells determined by SYTO9/PI staining (CFU/mL), c represents the number of culturable cells determined by TCBS plate counting (CFU/mL), and D indicates the VBNC incidence rate (%).

### 2.4. Preparation of Resuscitation Media

Five resuscitation media were prepared according to the method of Liu et al. [35], all based on TSB (Guangdong Huankai Biotechnology Co., Ltd., Guangzhou, China) with different supplements or treatments. The TSB control group and heat treatment group contained unsupplemented TSB. The TSB–sodium pyruvate group was supplemented with 2 mg/mL sodium pyruvate (Sangon Biotech Co., Ltd., Shanghai, China). The TSB–Tween 80 group was supplemented with 5% (*v*/*v*) Tween 80 (Sangon Biotech Co., Ltd., Shanghai, China). The TSB–signal molecule group was supplemented with C4-HSL, C6-HSL, C8-HSL, OHHL, and 3-oxo-C14-HSL (all from Sigma-Aldrich, St. Louis, MO, USA), each at a final concentration of 2 μmol/L. For preparation of 1 L of medium, the following amounts were added: 0.3985 mg C4-HSL, 0.4546 mg C6-HSL, 0.5107 mg C8-HSL, 0.4825 mg OHHL, and 0.6509 mg 3-oxo-C14-HSL. Media without signal molecules were sterilized by autoclaving at 121 °C for 15 min (DGL-50B, Jiangsu Dengguan Medical Equipment Co., Ltd., Changzhou, China), whereas media containing sodium pyruvate, Tween 80, or signal molecules were sterilized by filtration through 0.22 μm membrane filters. For the heat treatment group, bacterial cells were resuspended in TSB and subjected to heat shock at 72 °C for 10 s. All media were stored at 4 °C until use. The composition and preparation conditions of each group are summarized in Table S1 (Supplementary Material).

### 2.5. Resuscitation of VBNC-Vp

VBNC-Vp cells were harvested by centrifugation at 4 °C and 8000× *g* for 20 min (Avanti J-E, Beckman Coulter, Brea, CA, USA), washed twice with sterile phosphate-buffered saline (PBS), and concentrated by resuspension in a reduced volume of PBS to achieve a viable cell density of approximately 10^6^ CFU/mL (based on initial SYTO9/PI counts of 1.19 × 10^2^ CFU/mL). To minimize the possibility of unintended resuscitation during the concentration procedure, an aliquot of the concentrated cell suspension was immediately plated onto TCBS agar and incubated at 37 °C for 24 h. No colonies were detected, indicating that the concentrated cells remained non-culturable within the detection limit of the assay before the resuscitation experiments. The concentrated VBNC-Vp cells were then resuspended in the five resuscitation media described above. All samples were incubated at 37 °C with shaking at 160 r/min (ZQFW-70, Tianjin Laiboteri Instrument Co., Ltd., Tianjin, China) for 48 h. Bacterial growth was monitored by measuring the optical density at 600 nm (UV1810S spectrophotometer, Qingdao Juchuang Instrument Co., Ltd., Qingdao, China) at 3 h intervals, while culturable cell counts were determined by spreading onto TCBS plates at 24 h intervals. All treatments were performed in triplicate.

### 2.6. Morphological Observation by Scanning Electron Microscopy

Normal, VBNC, and resuscitated Vp cells (1 mL each) were harvested by centrifugation at 4 °C and 8000× *g* for 5 min (Avanti J-E, Beckman Coulter, Brea, CA, USA). The cell pellets were washed twice with PBS and fixed with 2.5% (*v*/*v*) glutaraldehyde (Sinopharm Chemical Reagent Co., Ltd., Shanghai, China) at 4 °C for 12 h. The fixed samples were then dehydrated through a graded ethanol series (30%, 50%, 70%, 80%, 90%, and 100%, Sinopharm Chemical Reagent Co., Ltd., Shanghai, China) with each step lasting 10 min. After dehydration, the samples were dried using the carbon dioxide critical point drying method, sputter-coated with gold for 1 min, and examined using a scanning electron microscope (JSM6360LV, JEOL, Tokyo, Japan) at an accelerating voltage of 20 kV. Ten random fields of view were selected for each sample for morphological analysis.

### 2.7. Cell Viability Staining

Cell viability of Vp was assessed using a SYTO9/PI dual-staining kit (Beyotime Biotechnology Co., Ltd., Shanghai, China). One milliliter of bacterial suspension from each treatment group was centrifuged at 4 °C and 8000× *g* for 5 min (Avanti J-E, Beckman Coulter, Brea, CA, USA). The supernatant was discarded, and the cell pellet was washed twice with sterile physiological saline and resuspended. SYTO9 and PI dyes (1.5 μL each) were added, followed by incubation in the dark for 15 min. After staining, a 10 μL aliquot of the suspension was placed on a glass slide, covered with a coverslip, and examined using a laser confocal scanning microscope (TCS SP8, Leica, Wetzlar, Germany). SYTO9 fluorescence was excited at 488 nm and detected at 500 nm (green, viable cells), whereas PI fluorescence was excited at 535 nm and detected at 617 nm (red, non-viable cells). Ten random fields of view were selected for each sample for analysis.

### 2.8. Intracellular ATP Concentration Assay

Intracellular ATP levels of normal, VBNC, and resuscitated Vp cells were determined using a bioluminescence-based ATP assay kit (Beyotime Biotechnology Co., Ltd., Shanghai, China). Cells were harvested by centrifugation, washed twice with 0.85% NaCl solution, and resuspended in sterile PBS. The cell density of each suspension was determined using SYTO9/PI staining combined with flow cytometry (CytoFLEX, Beckman Coulter, Brea, CA, USA), and all samples were diluted to an equal viable cell number (approximately 10^6^ cells/mL) based on the SYTO9-positive (live) cell counts. Then, 100 μL of the normalized bacterial suspension was mixed with 100 μL of BacTiter-GloTM reagent (Promega Corporation, Madison, WI, USA) and incubated at room temperature in the dark for 5 min. The chemiluminescence in relative luminescence units (RLU) was measured using a multimode microplate reader (Spark, TECAN, Männedorf, Switzerland). A standard curve was generated using a series of ATP standards (Beyotime Biotechnology Co., Ltd., Shanghai, China), and the absolute ATP concentration (μmol/L) was calculated accordingly. ATP concentrations were expressed as μmol per 10^6^ viable cells. All measurements were performed in triplicate.

### 2.9. Respiratory Chain Activity Assay

Respiratory chain activity Vp was evaluated using the 5-cyano-2,3-ditolyl tetrazolium chloride (CTC) staining method (CTC rapid staining kit, Beyotime Biotechnology Co., Ltd., Shanghai, China). Bacterial suspensions from each treatment group were adjusted to an OD_600_ of 0.5 ± 0.05. One milliliter of bacterial suspension was mixed with CTC formazan dye to a final concentration of 5 mmol/L and 1 μL of enhancer, followed by incubation at 37 °C in the dark for 30 min. After staining, the samples were centrifuged at 4 °C and 8000× *g* for 5 min (Avanti J-E, Beckman Coulter, Brea, CA, USA), washed twice with PBS, and resuspended. Fluorescence signals (excitation at 480 nm, emission at 630 nm) were detected using a flow cytometer (CytoFLEX, Beckman Coulter, Brea, CA, USA), with 10,000 cells analyzed per sample. Respiratory chain activity was expressed as mean fluorescence intensity per cell and normalized to that of normal cells (expressed as percentage).

### 2.10. Real-Time Quantitative PCR (RT-qPCR) for Key Gene Expression

Total RNA was extracted from Vp using the TRIzol method. RNA quality and concentration were evaluated using a spectrophotometer (UV1810S, Qingdao Juchuang Instrument Co., Ltd., Qingdao, China), with OD_260_/_280_ values ranging from 1.8 to 2.0, and integrity was verified by 1.2% agarose gel electrophoresis (DYY-6C, Beijing Liuyi Biotechnology Co., Ltd., Beijing, China). cDNA was synthesized using the PrimeScript™ RT reagent Kit (TaKaRa, Kusatsu, Shiga, Japan). Specific primers for opaR, atpA, soxR, katG, and the reference gene 16S rRNA [36] were designed and synthesized by Sangon Biotech Co., Ltd. (Shanghai, China) (primer sequences are listed in Table S2, Supplementary Material). Quantitative PCR was performed using TB Green^®^ Premix Ex Taq™ II (TaKaRa, Kusatsu, Shiga, Japan) on a LightCycler^®^ 480 II Real-Time PCR System (Roche, Basel, Switzerland). The amplification protocol consisted of an initial denaturation at 95 °C for 30 s, followed by 40 cycles of 95 °C for 5 s and 60 °C for 30 s. Melting curve analysis was conducted from 65 to 95 °C at a ramp rate of 0.5 °C/s to verify amplification specificity. Relative gene expression levels were calculated using the 2^−ΔΔCt^ method, with the normal group serving as the calibrator. All experiments were performed in triplicate, and results are presented as mean ± standard deviation.

### 2.11. Statistical Analysis

All experiments were performed in three independent biological replicates, and the results are presented as mean ± standard deviation (SD). Statistical analyses were conducted using SPSS 26.0 software (IBM, Armonk, NY, USA). Differences among multiple groups were evaluated by one-way analysis of variance (ANOVA), followed by Tukey’s honestly significant difference (HSD) post hoc test for pairwise comparisons. A *p* value < 0.05 was considered statistically significant. Graphical representations were generated using Origin 2021 software (OriginLab Corporation, Northampton, MA, USA).

## 3. Results

### 3.1. Induction of VBNC-Vp by Sodium Hypochlorite

**Figure 1:**
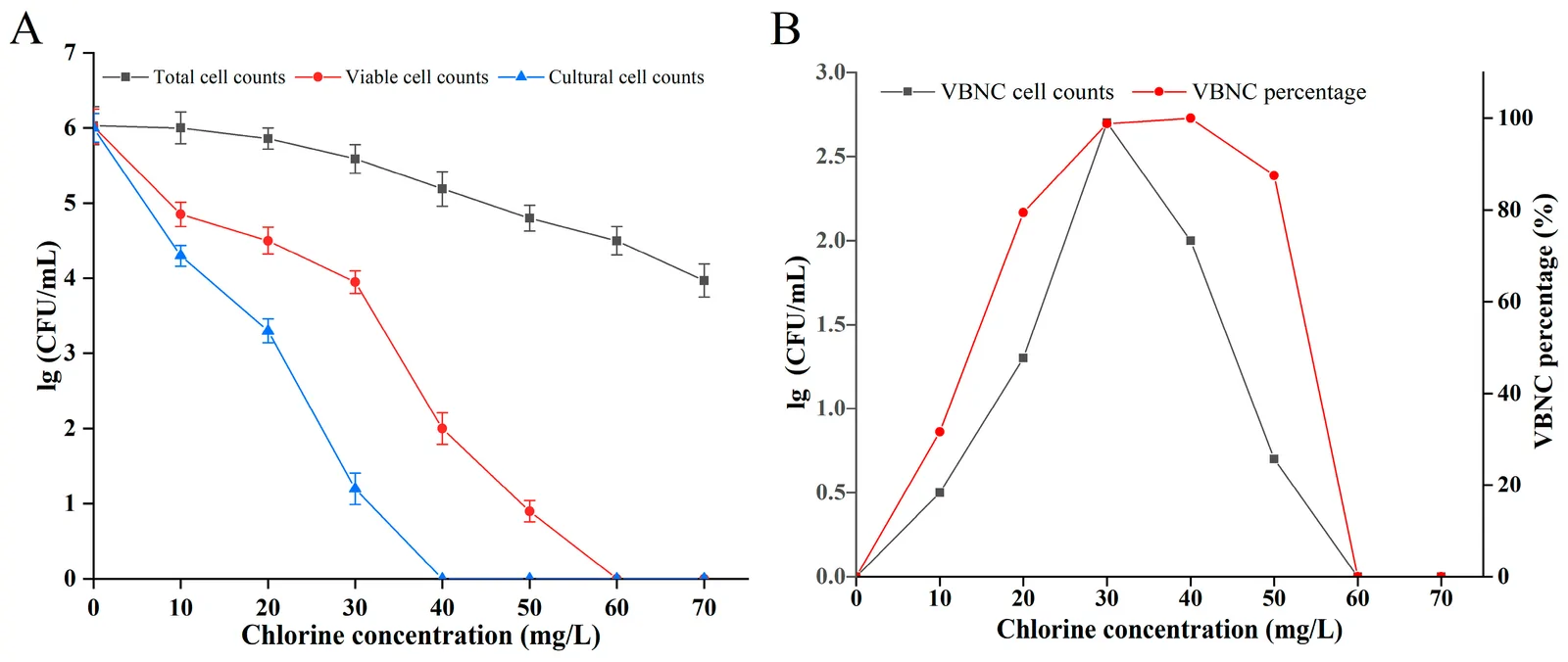
Induction of VBNC *Vibrio parahaemolyticus* by sodium hypochlorite. (**A**) Total, viable and culturable cell counts of Vp after treatment with varying available chlorine concentrations for 20 min, (**B**) VBNC cell count and VBNC incidence rate under the same treatment conditions. Data are presented as mean ± SD of three independent experiments.

Following exposure of Vp to NaClO at available chlorine concentrations of 10~70 mg/L for 20 min, culturable cell counts were determined using the TCBS plate count method. Total and viable cell counts were assessed using SYTO9/PI dual fluorescence staining combined with confocal laser scanning microscopy (Figure 1A). The limit of detection (LOD) for culturable cells by the plate count method was 10 CFU/mL (corresponding to 1 colony per plate when 100 µL of undiluted sample was plated). Culturable cell counts showed a concentration-dependent decline. The initial population was approximately 10^6^ CFU/mL. At 10 mg/L, a reduction of about 2 log units was observed. Between 10 and 20 mg/L, the decline slowed, with an additional reduction of approximately 1 log unit. A significant decrease (>2 log units) occurred between 20 and 30 mg/L, reducing counts to approximately 50 CFU/mL. At 40 mg/L, culturable cell counts fell below the limit of detection (LOD; <10 CFU/mL). In contrast, SYTO9/PI staining detected viable cells at (1.19 ± 0.12) × 10^2^ CFU/mL under this condition, with ≥90% of the cells displaying green fluorescence. Based on the combined criteria of loss of culturability, retention of membrane integrity, and basal metabolic activity, these findings suggest that the bacterial population had entered a VBNC state. However, as with all VBNC determinations based on viability staining, this classification is based on the standard operational definition and does not directly confirm the ability of cells to resume growth. This limitation was addressed by the subsequent resuscitation experiments (Section 3.2), which provide functional evidence supporting recovery from the VBNC state, although the possibility of outgrowth from a very small residual culturable subpopulation cannot be completely excluded. At 60 mg/L and 70 mg/L, both culturable and viable counts were below the LOD, indicating severe membrane damage and irreversible cell death.

VBNC cell counts and VBNC incidence rates were calculated using Equations (1) and (2) (Figure 1B). Total cell counts decreased as the available chlorine concentration increased. Within the range of 40–50 mg/L, the VBNC incidence rate approached 100%. However, VBNC cell counts were lower than those observed at 30 mg/L. At concentrations above 60 mg/L, both VBNC cell counts and VBNC incidence rates decreased to zero.

### 3.2. Effects of Different Resuscitation Systems on the Growth of VBNC-Vp

**Figure 2:**
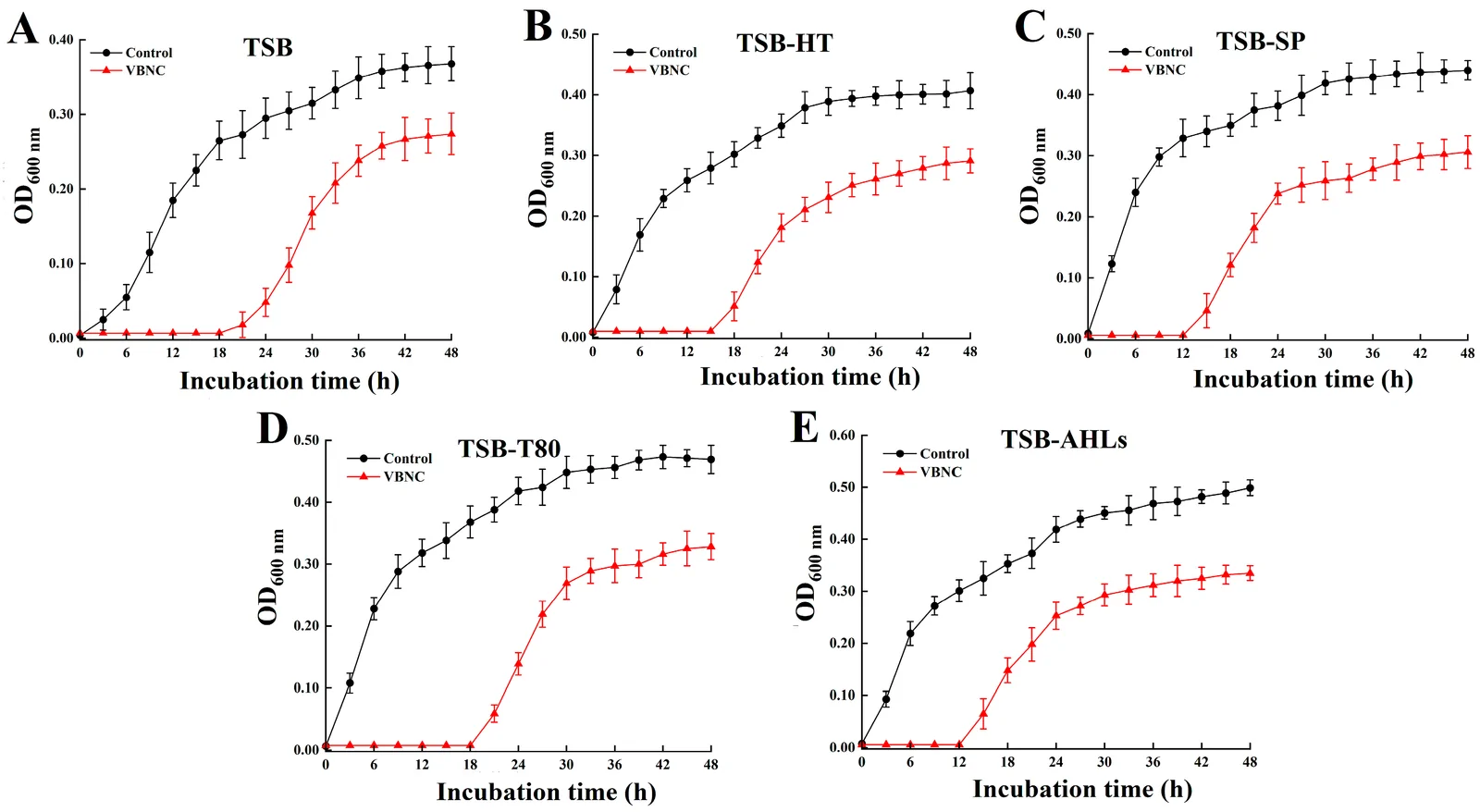
Effects of different resuscitation strategies on the growth kinetics of VBNC *Vibrio parahaemolyticus*. (**A**) TSB: control group cultured in tryptic soy broth, (**B**) TSB-HT: heat shock treatment at 72 °C for 10 s, (**C**) TSB-SP: TSB supplemented with 2 mg/mL sodium pyruvate, (**D**) TSB-T80: TSB supplemented with 5% Tween 80, (**E**) TSB-AHLs: TSB supplemented with five AHL signaling molecules (C4-HSL, C6-HSL, C8-HSL, OHHL, and 3-oxo-C14-HSL, each at 2 μmol/L). Data are presented as mean ± SD of three independent experiments.

VBNC-Vp cells were inoculated into five resuscitation systems: TSB (A), heat-treated TSB (B), TSB supplemented with sodium pyruvate (C), TSB supplemented with Tween 80 (D), and TSB supplemented with signal molecules (E). Cultures were incubated at 37 °C with shaking. Resuscitation was evaluated by monitoring changes in OD_600_ over time (Figure 2). The TSB group exhibited the slowest resuscitation rate, with a lag phase of 21 h and the lowest final biomass among all groups (Figure 2A). The heat-treated TSB group showed a slightly earlier onset of resuscitation, with a lag phase of 18 h. However, the improvement remained limited (Figure 2B). The sodium pyruvate and Tween 80 groups entered the logarithmic phase at 15 h and 21 h, respectively. The sodium pyruvate group exhibited a shorter lag phase (Figure 2C,D). The signal molecule group demonstrated the most pronounced resuscitation effect. Cells entered the logarithmic phase at 15 h and exhibited the fastest growth rate. The final OD_600_ value was significantly higher than those of the other treatment groups (*p* < 0.05). It approached the level of the normal control group (Figure 2E). These results indicate that exogenous addition of quorum sensing signal molecules effectively enhanced metabolic activity in VBNC-Vp. The lag phase was substantially shortened, and resuscitation efficiency was significantly enhanced.

### 3.3. Colony Morphology of Resuscitated Vp

**Figure 3:**
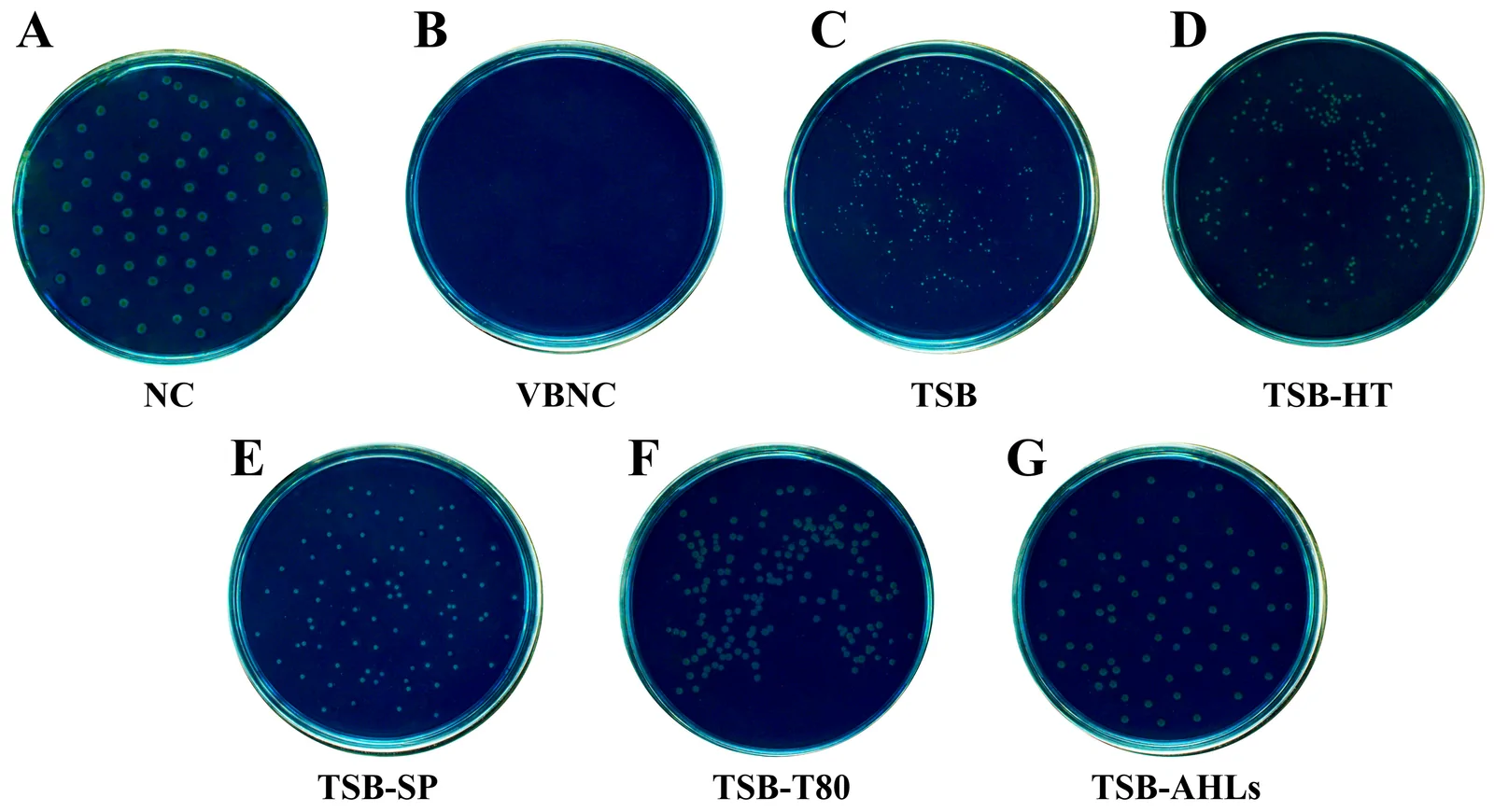
Effects of different resuscitation strategies on the colony morphology of VBNC *Vibrio parahaemolyticus* on TCBS agar plates. (**A**) NC: normal cell group, (**B**) VBNC: VBNC cell group, (**C**) TSB: cells resuscitated in tryptic soy broth, (**D**) TSB-HT: heat shock treatment at 72 °C for 10 s, (**E**) TSB-SP: TSB supplemented with 2 mg/mL sodium pyruvate, (**F**) TSB-T80: TSB supplemented with 5% Tween 80, (**G**) TSB-AHLs: TSB supplemented with five AHL signaling molecules (C4-HSL, C6-HSL, C8-HSL, OHHL, and 3-oxo-C14-HSL, each at 2 μmol/L).

The colony morphology of VBNC-Vp after different resuscitation treatments was evaluated on TCBS agar plates (Figure 3). Colonies in the normal control group showed typical characteristics, appearing blue-green, circular, smooth-edged, and approximately 2~3 mm in diameter (Figure 3A). No colonies were observed in the VBNC group (Figure 3B), indicating the loss of culturability. The TSB group produced a limited number of small and pale colonies (Figure 3C), indicating the low resuscitation efficiency of conventional TSB. The TSB-heat treatment group showed a slight increase in colony number, but the overall recovery remained low (Figure 3D). Both the TSB-sodium pyruvate and TSB-Tween 80 groups exhibited a significant increase in colony count, with larger colonies distributed evenly across the plates (Figure 3E,F). Notably, the TSB-AHLs group formed the highest colony count, with dense distribution, typical coloration, and morphology closely resembling that of the normal control group (Figure 3G). These results further demonstrate that the quorum sensing signal molecule-based system efficiently enhances the recovery of culturability in VBNC-Vp.

### 3.4. Ultrastructure of Resuscitated Vp Cells

**Figure 4:**
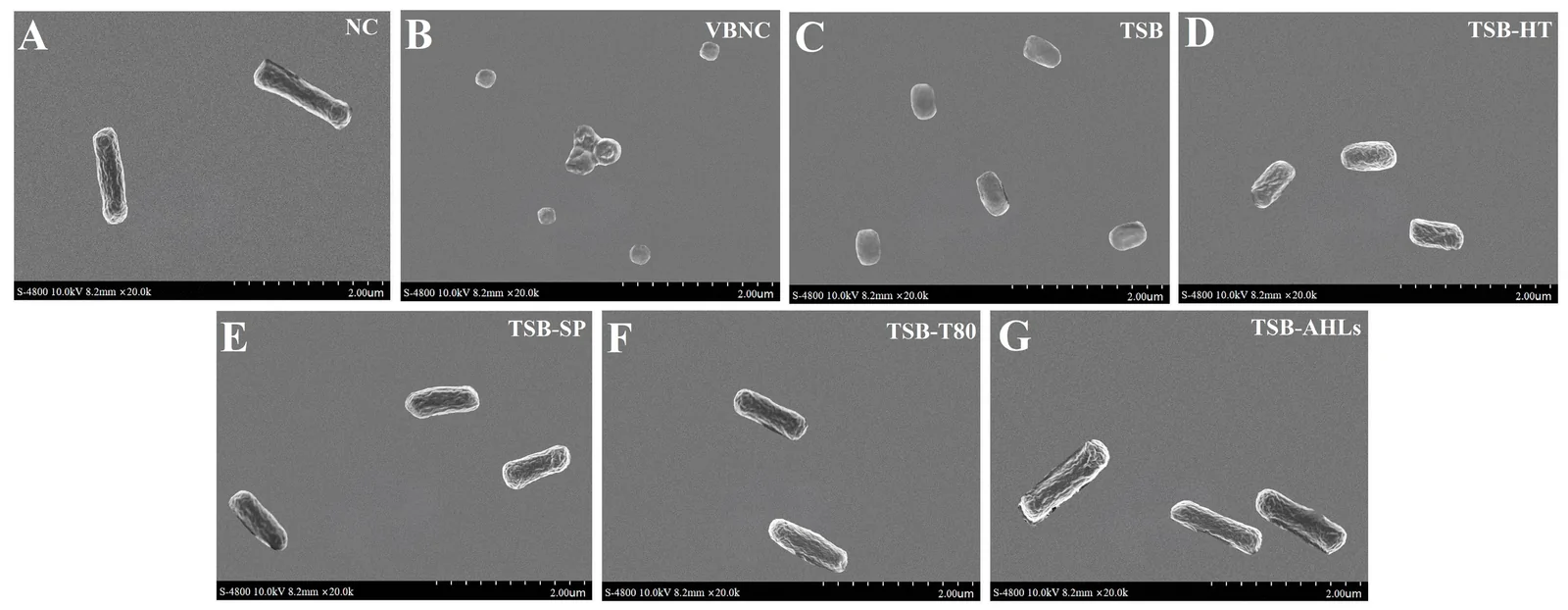
Effects of different resuscitation strategies on the cell morphology of VBNC *Vibrio parahaemolyticus* observed under scanning electron microscopy (SEM). (**A**) NC: normal cell group, (**B**) VBNC: VBNC cell group, (**C**) TSB: cells resuscitated in tryptic soy broth, (**D**) TSB-HT: heat shock treatment at 72 °C for 10 s, (**E**) TSB-SP: TSB supplemented with 2 mg/mL sodium pyruvate, (**F**) TSB-T80: TSB supplemented with 5% Tween 80, (**G**) TSB-AHLs: TSB supplemented with five AHL signaling molecules (C4-HSL, C6-HSL, C8-HSL, OHHL, and 3-oxo-C14-HSL, each at 2 μmol/L).

Cellular morphology under different treatment conditions was examined using scanning electron microscopy (Figure 4). Cells in the normal control group exhibited a typical short-rod shape, with a smooth and plump surface, clear boundaries, and random distribution (Figure 4A). Following induction with 40 mg/L sodium hypochlorite, cells in the VBNC group showed pronounced morphological alterations. Most cells transitioned from a rod-shaped to a coccoid or ellipsoid morphology, accompanied by severe surface wrinkling, depressions, and increased roughness; aggregation was also observed in some cases (Figure 4B), indicating substantial structural remodeling under NaClO stress. After resuscitation in TSB alone, partial recovery was observed, with some cells regaining a short rod-shaped morphology; however, surface irregularities persisted (Figure 4C). The TSB-heat treatment group showed further morphological improvement, although the extent of recovery remained limited (Figure 4D). In contrast, both the TSB-SP and TSB-T80 groups demonstrated a notable restoration of rod-shaped morphology, with smoother cell surfaces and reduced aggregation (Figure 4E,F). Notably, the TSB-AHLs group exhibited the most pronounced recovery, with most cells restoring a regular short rod-shaped morphology and smooth surface features, closely resembling those of the normal control group in most respects. However, a few cells still displayed minor surface irregularities (Figure 4G). To quantitatively assess morphological recovery, cell length, width, and aspect ratio (length/width) were measured from SEM images using ImageJ (version 1.54g) software. As shown in Table 1, VBNC cells exhibited significantly reduced cell length (0.82 ± 0.15 μm) and aspect ratio (1.45 ± 0.18) compared with normal cells (1.68 ± 0.22 μm and 2.68 ± 0.31, respectively), reflecting the characteristic coccoid transition. The TSB-AHLs group showed the greatest morphological recovery, with cell length (1.52 ± 0.19 μm) and aspect ratio (2.45 ± 0.22) approaching those of normal cells, whereas the TSB and TSB-HT groups showed only partial recovery (cell length: 1.15 ± 0.17 μm and 1.21 ± 0.16 μm, respectively; aspect ratio: 1.88 ± 0.20 and 1.95 ± 0.18, respectively). These quantitative findings are consistent with the qualitative SEM observations and suggest that AHL supplementation was associated with greater morphological recovery than the other resuscitation treatments evaluated. These results indicate that the signal molecule-based resuscitation system was associated with improved cellular morphology and promotes the restoration of cellular structural integrity, although a very small subset of cells may not achieve full morphological recovery under the conditions tested.

### 3.5. Cell Viability of Resuscitated Vp

**Figure 5:**
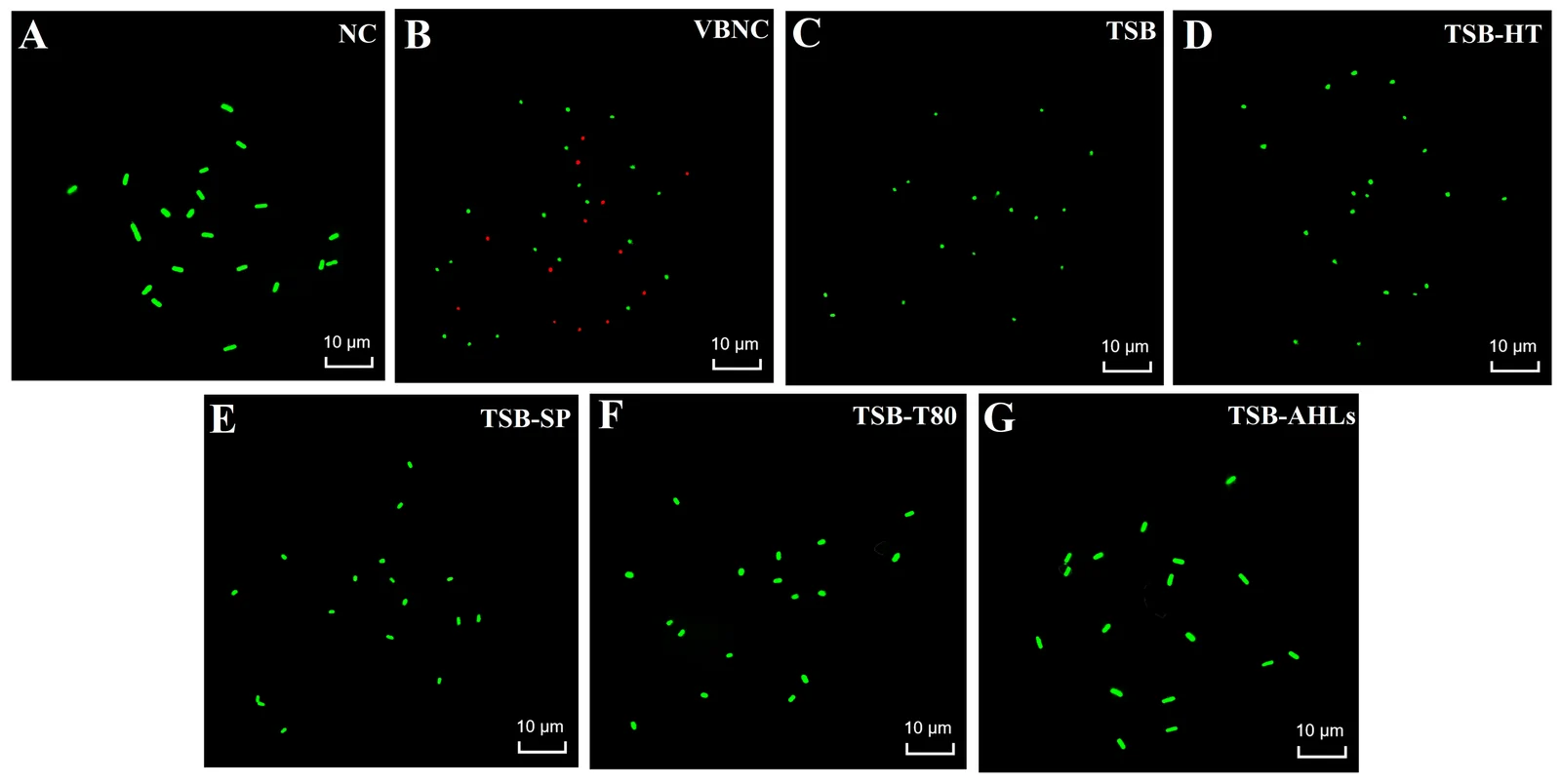
Effects of different resuscitation strategies on the cell viability of VBNC *Vibrio parahaemolyticus* assessed by SYTO9/PI dual fluorescence staining. (**A**) NC: normal cell group, (**B**) VBNC: VBNC cell group, (**C**) TSB: cells resuscitated in tryptic soy broth, (**D**) TSB-HT: heat shock treatment at 72 °C for 10 s, (**E**) TSB-SP: TSB supplemented with 2 mg/mL sodium pyruvate, (**F**) TSB-T80: TSB supplemented with 5% Tween 80, (**G**) TSB-AHLs: TSB supplemented with five AHL signaling molecules (C4-HSL, C6-HSL, C8-HSL, OHHL, and 3-oxo-C14-HSL, each at 2 μmol/L). SYTO9 stains viable cells with green fluorescence, whereas propidium iodide (PI) labels membrane-compromised cells with red fluorescence. Scale bar = 10 μm (applicable to all panels).

Cell membrane integrity and viability of VBNC-Vp after different resuscitation treatments were evaluated using SYTO9/PI dual fluorescence staining in combination with laser confocal scanning microscopy (Figure 5). Cells in the normal control group exhibited strong and uniformly distributed green fluorescence with a typical short rod-shaped morphology, and no red fluorescence was detected (Figure 5A), indicating intact membranes and high viability. In the VBNC group, most cells appeared coccoid or irregular in shape, accompanied by significantly reduced green fluorescence; a proportion of cells displayed red fluorescence (Figure 5B), suggesting membrane damage and decreased viability induced by NaClO treatment. After resuscitation in TSB alone, an increase in green-fluorescent cells was observed, with no evident red fluorescence; however, the overall recovery remained limited (Figure 5C). The TSB-heat treatment group showed a further enhancement in green fluorescence intensity compared with the TSB group (Figure 5D). In contrast, the TSB-SP, TSB-T80, and TSB-AHLs groups exhibited a substantial increase in the number of green-fluorescent cells, accompanied by a gradual restoration of the typical short rod-shaped morphology (Figure 5E–G). Among these treatments, the TSB-AHLs group demonstrated the highest fluorescence intensity and cell density, closely resembling the normal control group. These findings indicate that exogenous AHL supplementation effectively promotes the recovery of membrane integrity and metabolic activity in VBNC-Vp.

### 3.6. Intracellular ATP Levels in Resuscitated Vp

**Figure 6:**
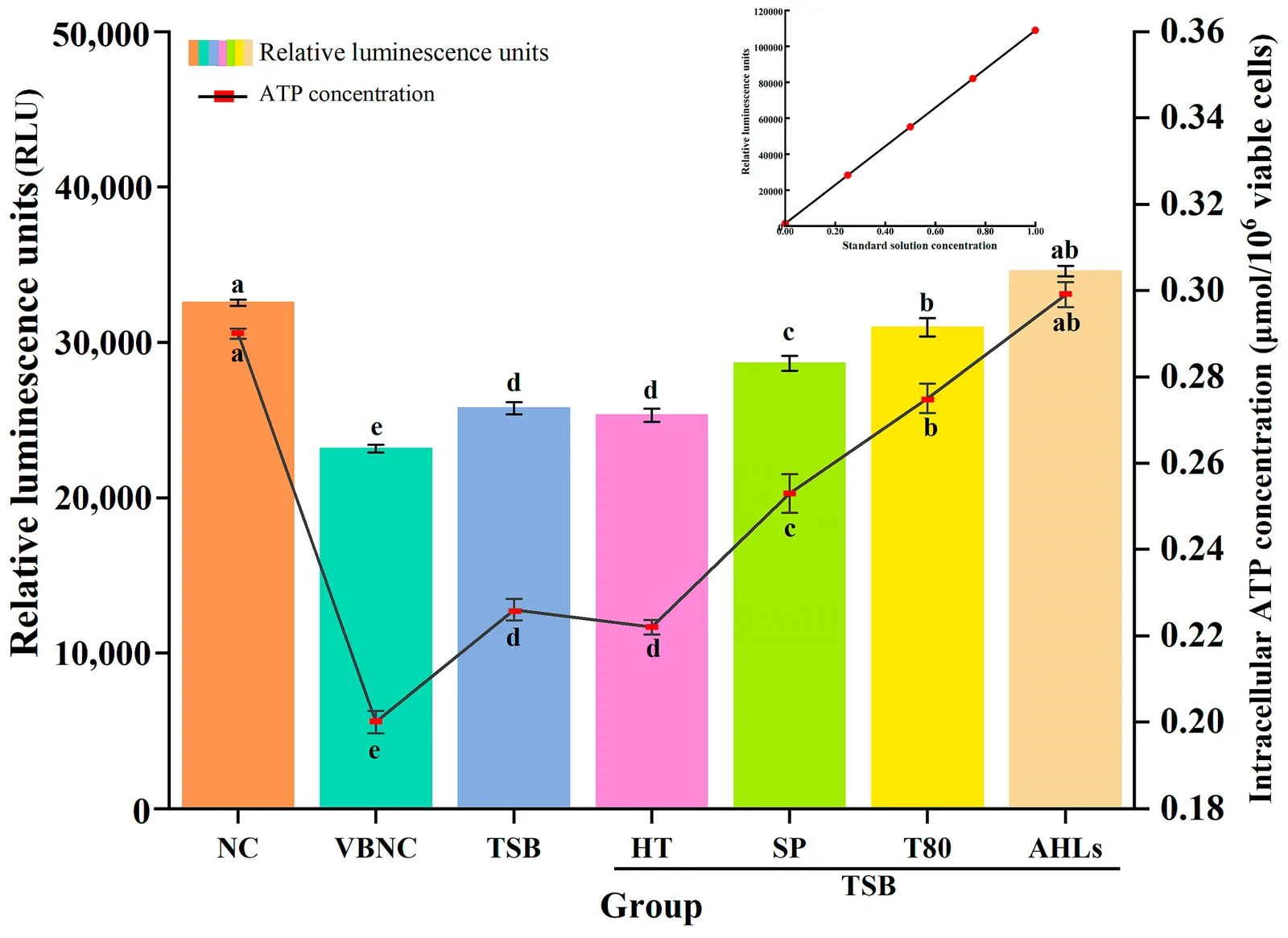
Effects of different resuscitation strategies on the intracellular ATP concentration of VBNC *Vibrio parahaemolyticus*. NC: normal cell group, VBNC: VBNC cell group, TSB: cells resuscitated in tryptic soy broth, HT: heat shock treatment at 72 °C for 10 s, SP: TSB supplemented with 2 mg/mL sodium pyruvate, T80: TSB supplemented with 5% Tween 80, AHLs: TSB supplemented with five AHL signaling molecules (C4-HSL, C6-HSL, C8-HSL, OHHL, and 3-oxo-C14-HSL, each at 2 μmol/L). Data are presented as mean ± SD from three independent experiments. Different lowercase letters (a–e) indicate significant differences between groups (*p* < 0.05).

Intracellular ATP concentration was used as an indicator of cellular metabolic activity. Chemiluminescence intensity and corresponding ATP levels were determined for each treatment group (Figure 6). The normal control group exhibited a chemiluminescence value of 32,500 RLU, corresponding to an ATP concentration of (0.29 ± 0.0012) μmol/L. In contrast, the VBNC group showed a reduced chemiluminescence value of 24,000 RLU and a significantly lower ATP concentration of (0.19 ± 0.0026) μmol/L (*p* < 0.05), representing a decrease of approximately 34.5%, indicative of suppressed metabolic activity in the VBNC state.

After resuscitation in TSB alone, the chemiluminescence value increased to 28,000 RLU, with a corresponding ATP concentration of (0.226 ± 0.0025) μmol/L. The TSB-HT group exhibited a chemiluminescence value of 27,500 RLU and an ATP concentration of (0.224 ± 0.0017) μmol/L, indicating limited metabolic recovery. In contrast, the TSB-SP and TSB-T80 groups demonstrated higher chemiluminescence values of 32,000 and 33,000 RLU, respectively, with corresponding ATP concentrations of (0.24 ± 0.0035) μmol/L and (0.26 ± 0.0027) μmol/L. These values were significantly greater than those observed in the TSB group (*p* < 0.05), indicating enhanced metabolic restoration. Notably, the TSB-AHLs group exhibited the most pronounced increase, with a chemiluminescence value of 34,000 RLU and an ATP concentration of (0.28 ± 0.0029) μmol/L.

This level was not statistically different from that of the normal control group (*p* > 0.05) and slightly exceeded it. These results indicate that exogenous AHLs effectively stimulate cellular energy metabolism in VBNC-Vp, promoting ATP synthesis and thereby facilitating the resuscitation process.

### 3.7. Respiratory Chain Activity of Resuscitated Vp

**Figure 7:**
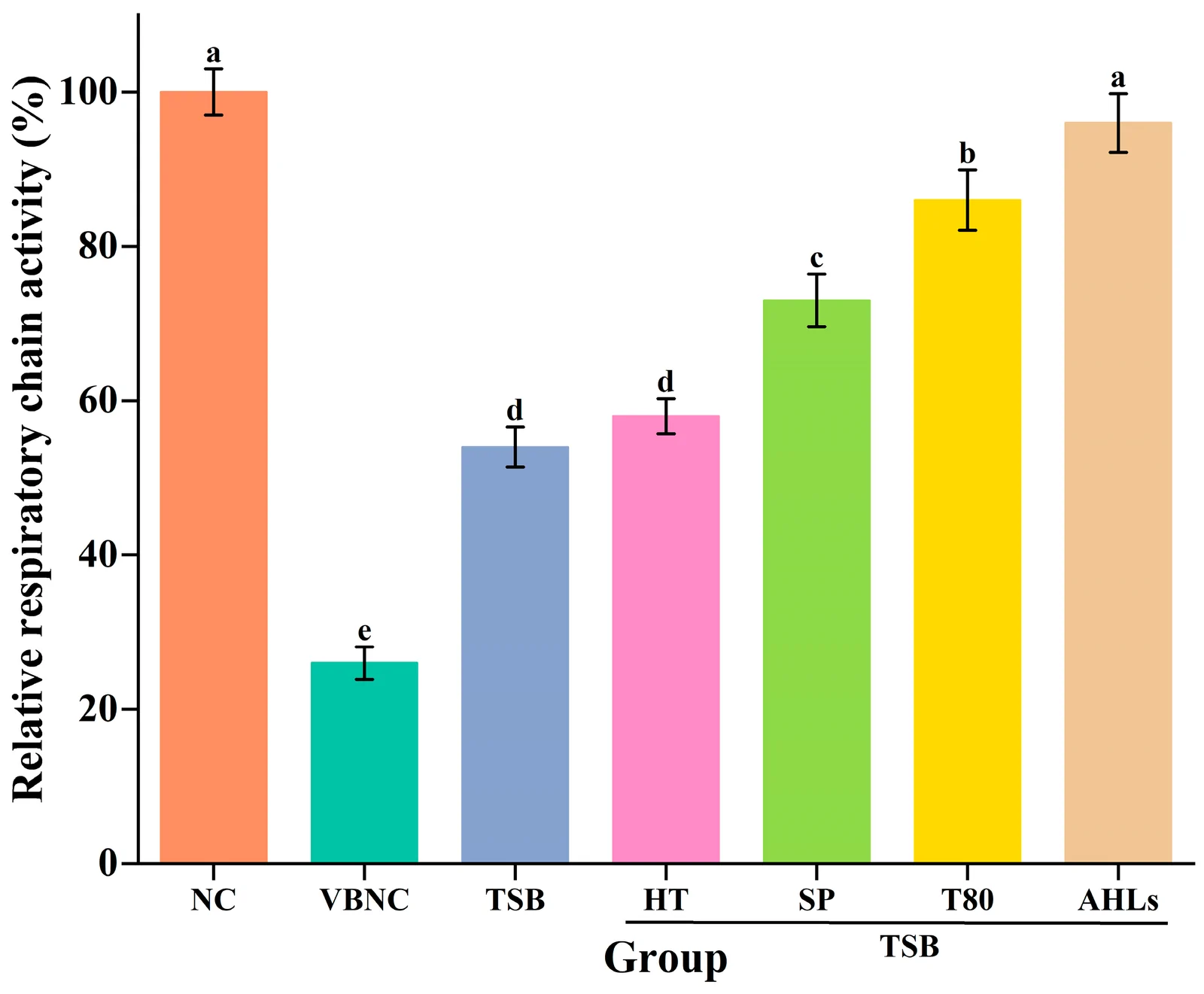
Effects of different resuscitation strategies on the respiratory chain activity of VBNC *Vibrio parahaemolyticus*. NC: normal cell group, VBNC: VBNC cell group, TSB: cells resuscitated in tryptic soy broth, HT: heat shock treatment at 72 °C for 10 s, SP: TSB supplemented with 2 mg/mL sodium pyruvate, T80: TSB supplemented with 5% Tween 80, AHLs: TSB supplemented with five AHL signaling molecules (C4-HSL, C6-HSL, C8-HSL, OHHL, and 3-oxo-C14-HSL, each at 2 μmol/L). Data are presented as mean ± SD of three independent experiments. Different lowercase letters indicate significant differences between groups (*p* < 0.05).

Respiratory chain activity of VBNC-Vp after different resuscitation treatments was evaluated using CTC staining coupled with flow cytometry, with the activity of the normal control group defined as 100% (Figure 7). The VBNC group exhibited a marked reduction in respiratory chain activity to (26.17 ± 2.08)% of the normal control level (*p* < 0.05), indicating that NaClO exposure severely impaired respiratory enzyme function. The TSB and TSB-HT groups showed partial recovery, with respiratory chain activities of (54.45 ± 2.31)% and (58.67 ± 2.52)% of the control level, respectively, reflecting limited restoration. In contrast, the TSB-SP and TSB-T80 groups exhibited enhanced recovery, with activities reaching (73.32 ± 2.78)% and (86.89 ± 2.64)%, respectively, both significantly higher than that of the TSB group (*p* < 0.05). Notably, the TSB-AHLs group showed the most pronounced recovery, achieving a respiratory chain activity of (96.63 ± 3.02)% of the normal control level, which was significantly higher than all other treatment groups (*p* < 0.05).

These results further demonstrate, from the perspective of energy metabolism, that supplementation with AHLs effectively promotes the reactivation of respiratory chain enzymes in VBNC-Vp, facilitating the transition from a dormant to a metabolically active state and highlighting its strong potential for enhancing resuscitation efficiency.

### 3.8. Changes in Expression of Key Genes During Resuscitation

**Figure 8:**
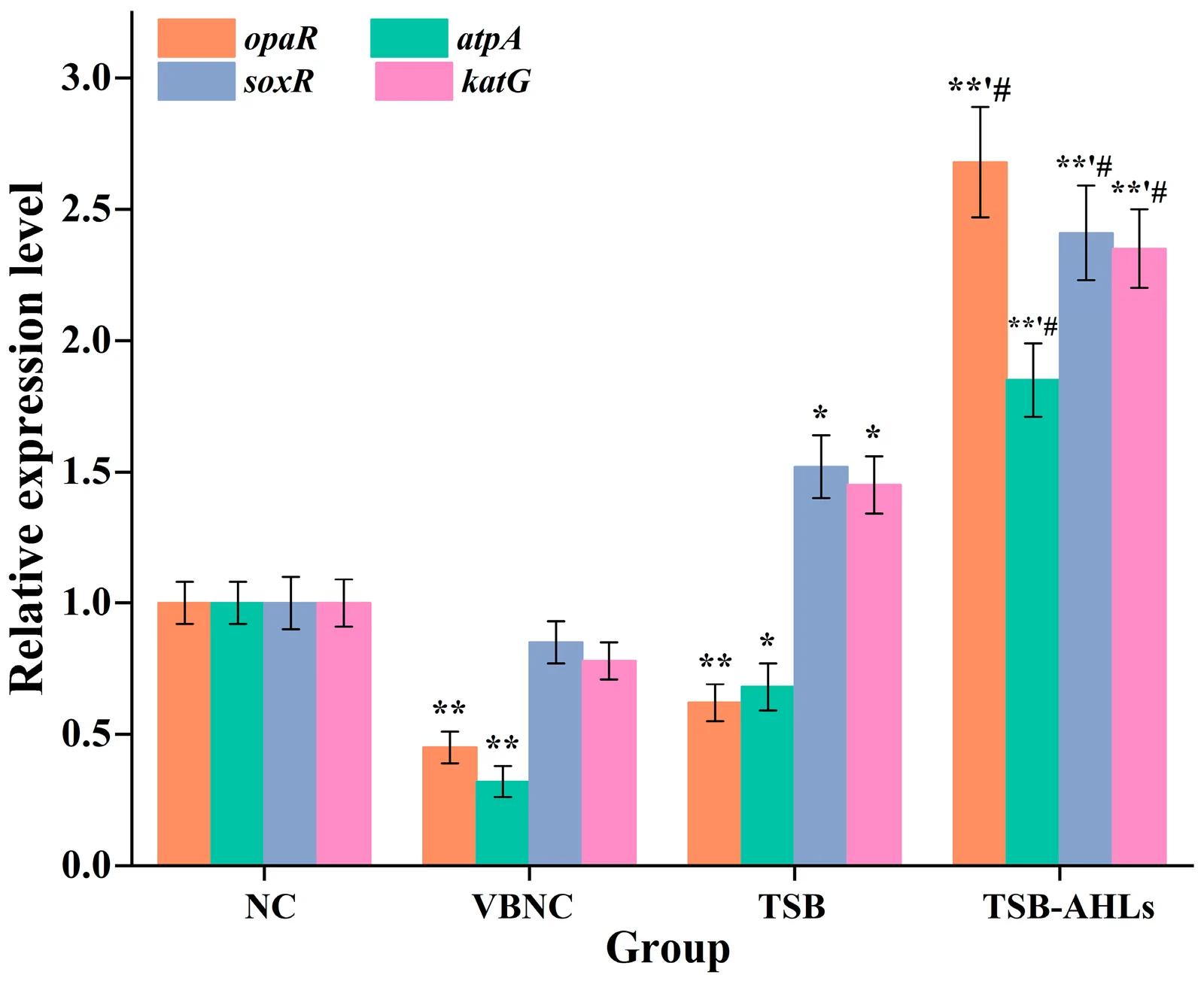
Relative expression levels of opaR, atpA, soxR, and katG genes in *Vibrio parahaemolyticus* under different treatment conditions. NC: normal cell group; VBNC: VBNC cell group; TSB: cells resuscitated in tryptic soy broth; TSB-AHLs: TSB supplemented with five AHL signaling molecules (C4-HSL, C6-HSL, C8-HSL, OHHL, and 3-oxo-C14-HSL, each at 2 μmol/L). Data are presented as mean ± SD of three independent experiments. * indicates a significant difference compared with the normal group (* *p* < 0.05, ** *p* < 0.01), and # indicates a significant difference compared with the TSB group (# *p* < 0.05).

To investigate the molecular mechanism underlying the efficient resuscitation observed in the TSB-AHLs group, the relative expression levels of selected functional genes were analyzed by RT-qPCR in the normal control, VBNC, TSB, and TSB-AHLs groups (Figure 8). The genes examined included the quorum sensing regulator (opaR), the energy metabolism-related gene (atpA), the oxidative stress regulator (soxR), and the catalase-encoding gene (katG).

In terms of quorum sensing regulation, opaR expression in the VBNC group was significantly downregulated to 0.45-fold of the normal control level (*p* < 0.01), indicating that NaClO stress severely suppressed transcription of this core quorum sensing regulator. After resuscitation with TSB alone, opaR expression recovered to 0.62-fold, but remained significantly lower than the normal control level (*p* < 0.01). In contrast, the TSB-signal molecule group exhibited a marked upregulation of opaR expression to 2.68-fold, which was significantly higher than both the TSB group (*p* < 0.05) and the normal control group (*p* < 0.01), demonstrating that exogenous AHLs effectively activated the Vp quorum sensing system and upregulated opaR expression.

With respect to energy metabolism, atpA expression in the VBNC group was significantly downregulated to 0.32-fold of the normal control level (*p* < 0.01), consistent with the marked decrease in ATP concentration in VBNC cells, indicating that NaClO stress severely inhibited ATP synthase transcription. Following resuscitation with TSB alone, atpA expression recovered to 0.68-fold but remained significantly lower than the normal control level (*p* < 0.05). In contrast, the TSB-AHLs group showed upregulation of atpA expression to 1.85-fold, which was significantly higher than both the TSB group (*p* < 0.05) and the normal control group (*p*< 0.01). This increase was accompanied by the restoration of ATP concentration to a level comparable to that of the normal control, suggesting that exogenous AHL supplementation was associated with increased ATP production and enhanced atpA expression.

Regarding the oxidative stress response, soxR and katG expression levels in the VBNC group were 0.85-fold and 0.78-fold of the normal control levels, respectively, with no statistically significant differences (*p* > 0.05 for both genes compared with the normal control group). After resuscitation in TSB, soxR and katG expression increased to 1.52-fold and 1.45-fold, respectively (*p* < 0.05). Notably, the TSB-AHLs group showed a more pronounced upregulation, with soxR and katG reaching 2.41-fold and 2.35-fold, respectively (*p* < 0.01), and these values were significantly higher than those in the TSB group (*p* < 0.05). These results indicate that exogenous AHL supplementation effectively activated the antioxidant defense system of VBNC-Vp by upregulating soxR and katG expression, thereby enhancing catalase production and improving cellular resistance to oxidative stress induced by NaClO.

## 4. Discussion

Sodium hypochlorite (NaClO) is widely employed as a disinfectant in seafood processing due to its broad-spectrum bactericidal activity and cost-effectiveness. However, increasing evidence indicates that sublethal or inappropriate concentrations of NaClO may induce various foodborne pathogens to enter the VBNC state [37]. In the present study, culturable cell counts decreased in a concentration-dependent manner with increasing levels of available chlorine. The initial culturable count was approximately 10^6^ CFU/mL. Exposure to 10 mg/L available chlorine resulted in a reduction of approximately 2 log units. Between 10 and 20 mg/L, the rate of decline slowed, with a reduction of approximately 1 log unit. In contrast, a sharp decrease of more than 2 log units was observed between 20 and 30 mg/L, with culturable counts declining to approximately 50 CFU/mL. At 40 mg/L available chlorine, culturable cells became undetectable, whereas SYTO9/PI staining indicated the presence of viable cells at (1.19 ± 0.12) × 10^2^ CFU/mL (Figure 1A). These results suggest that the subpopulation had entered a VBNC-like state. Within the range of 30–40 mg/L, the VBNC incidence rate approached 100%, although VBNC cell counts were lower than at 30 mg/L (Figure 1B). This reduction may be attributed to increased cellular damage or death of pre-existing VBNC cells at higher NaClO concentrations. At available chlorine concentrations of 60 mg/L or higher, total, viable, and culturable counts all fell below detectable limits, indicating severe and irreversible membrane damage leading to complete cell death.

These findings are consistent with previous reports. For example, Wang et al. [38] demonstrated that exposure to 45 mg/L available chlorine induced *Salmonella enteritidis* to enter the VBNC state, suggesting that diverse foodborne pathogens may exhibit similar responses to NaClO-induced stress. Hu et al. [39] found that *Yersinia enterocolitica* entered the VBNC state after exposure to 50 mg/L available chlorine for 12 min, whereas Zhao et al. [40] observed the formation of dormant cells in *Pseudomonas aeruginosa* under treatment with 60 mg/L NaClO. In the present study, Vp entered the VBNC state after 20 min of treatment with 40 mg/L available chlorine, suggesting a potentially higher sensitivity to NaClO compared with the other species mentioned above. Several factors may explain this apparent difference. First, the initial bacterial density in our study (10^6^ CFU/mL) was lower than that used in some previous studies (e.g., 10^7^–10^8^ CFU/mL), and a lower initial cell density may require a lower chlorine concentration to achieve complete loss of culturability due to reduced protective effects from cell aggregation or biofilm-like structures. Second, strain-specific variations in cell wall composition and outer membrane permeability could influence chlorine susceptibility. Third, differences in oxidative stress defense mechanisms, such as the expression and activity of catalase or superoxide dismutase, may affect the ability to tolerate NaClO-induced damage. These possibilities are consistent with the observation by Jacob et al. [41] that bacterial tolerance to oxidative stress varies considerably among species and even among strains of the same species. However, at concentrations exceeding 60 mg/L, all of the aforementioned bacterial strains were completely inactivated, indicating that sufficiently high levels of NaClO can effectively eliminate cells and prevent the formation of VBNC state. Accordingly, maintaining available chlorine concentrations above 60 mg/L during seafood processing may help minimize VBNC formation and subsequent resuscitation, thereby reducing the risk of false-negative detection [42].

The resuscitation of VBNC-state bacteria is regulated by various environmental factors, among which nutrient availability plays a pivotal role. In the present study, conventional TSB was able to partially resuscitate VBNC-Vp; however, the overall efficiency was limited, as reflected by a prolonged lag phase of 21 h and low final biomass (Figure 2A), consistent with previous findings. This limited recovery may be attributed to the reduced metabolic activity of VBNC cells, which constrains their ability to rapidly respond to conventional nutrient conditions [43]. To enhance resuscitation efficiency, sodium pyruvate and Tween 80 were supplemented into TSB (Figure 2C,D). Sodium pyruvate shortened the resuscitation lag phase to 15 h and significantly increased both ATP concentration and respiratory chain activity compared with the TSB group (Figure 6 and Figure 7). As a reactive oxygen species (ROS) scavenger, sodium pyruvate mitigates NaClO-induced oxidative damage and contributes to the restoration of cell membrane integrity and respiratory enzyme function. Yang et al. [44] similarly reported that sodium pyruvate significantly promoted the resuscitation of VBNC *Salmonella*, attributing this effect to its dual role as a carbon source, entering directly into the tricarboxylic acid cycle to support energy production, and as a scavenger of free radicals that reduces oxidative stress. Tween 80, a non-ionic surfactant, reduces surface tension, increases cell membrane permeability, and promotes nutrient uptake, thereby facilitating resuscitation. Wei et al. [45] demonstrated that nutrient broth supplemented with 5% Tween 80 effectively resuscitated VBNC *E. coli*, with higher final cell yields compared to sodium pyruvate under the same basal conditions. The present findings are consistent with this observation (Figure 2C,D). However, the onset of resuscitation in the Tween 80 group (21 h) was delayed relative to that in the sodium pyruvate group (15 h), suggesting that sodium pyruvate exerts a more immediate effect, likely through the direct alleviation of oxidative stress.

Quorum sensing (QS) is a regulatory mechanism by which bacteria produce and detect autoinducers to coordinate gene expression in a cell density-dependent manner. In Gram-negative bacteria, AHLs serve as the principal QS signaling molecules, regulating a wide range of physiological processes and metabolic activities [46]. Increasing evidence suggests that QS signaling molecules play an important role in the resuscitation of VBNC-state bacteria. Ayrapetyan et al. [19] found that exogenous supplementation with AHLs, including C4-HSL, C6-HSL, C8-HSL, OHHL, and 3-oxo-C14-HSL, significantly promoted the resuscitation of dormant Vibrio species at concentrations ranging from 0.2 to 2 μmol/L. Consistent with these findings, a concentration of 2 μmol/L for each AHL was employed in the present study, resulting in a more pronounced resuscitation effect in Vp, likely due to the combined application of all five AHLs rather than individual compounds. Liao et al. [20] similarly demonstrated that activation of the QS system reversed the VBNC state of *Salmonella typhimurium* by triggering catalase expression. In the present study, supplementation of TSB with the five AHLs enabled VBNC-Vp to enter the logarithmic growth phase at 15 h, accompanied by the highest colony count and most typical morphology. Cells recovered a regular short rod-shaped structure, while intracellular ATP levels increased significantly and respiratory chain activity reached (96.63 ± 3.02)% of the normal control level, significantly exceeding those of all other treatment groups (Figure 6 and Figure 7). These results suggest that exogenous AHL supplementation may facilitate the recovery of VBNC *V. parahaemolyticus* by promoting a quorum sensing-related signaling environment conducive to metabolic reactivation and cellular recovery. However, the present study demonstrates an association rather than a direct causal mechanism. Because a mixture of five AHLs was used, it was not possible to determine which individual signaling molecule(s) were primarily responsible for the observed effects or whether synergistic interactions among the AHLs contributed to the response. Future studies using individual AHLs and defined combinations, together with functional validation, are needed to elucidate the specific roles and underlying mechanisms of each signaling molecule in VBNC recovery.

The efficient resuscitation observed in the TSB-AHLs group is likely attributable to the specific interaction between AHLs and the QS receptor protein OpaR, which activates the downstream signal transduction cascade [47]. Wu et al. [48] reported that AHLs bind to the LuxR protein in *Vibrio* species, forming a complex that upregulates ATP synthase expression, increases intracellular ATP levels, and thereby enhances ATP-dependent processes such as DNA replication and translation, ultimately promoting cell division. This mechanism is consistent with the substantial increase in ATP levels observed in the TSB-AHLs group in the present study. In addition, QS signaling molecules may modulate toxin–antitoxin regulatory networks, enhance the expression of antioxidant enzymes, relieve constraints on cell division, and restore respiratory chain function, thereby facilitating the transition of VBNC cells from a dormant to an active state [49,50]. The significant recovery of respiratory chain activity observed in the TSB-AHLs group further supports this mechanism from an energy metabolism perspective.

To further validate the proposed mechanisms, RT-qPCR analysis was performed to determine the relative expression levels of the QS regulator (opaR), the energy metabolism-related gene (atpA), and the antioxidant defense-related genes (soxR and katG) across treatment groups (Figure 8). The results showed that opaR expression in the TSB-AHLs group was significantly upregulated to 2.68-fold relative to the normal control, whereas expression in the TSB group remained suppressed (0.62-fold, which is still significantly below the normal control level), indicating only partial recovery without reaching normal levels. This finding suggests that exogenous AHLs may activate the Vp QS system, whereas nutrient supplementation alone appears insufficient to elicit a comparable response. However, increased opaR expression alone does not confirm activation of the QS system or establish a direct causal role of QS in the recovery of VBNC cells. Moreover, atpA expression was upregulated by 1.85-fold in the TSB-AHLs group, consistent with the recovery of ATP concentration to normal levels to near-normal values. These findings suggest that exogenous AHL supplementation is associated with increased atpA expression and ATP production; however, the underlying mechanism requires further investigation. In addition, the expression levels of soxR and katG in the TSB-AHLs group were increased by 2.41-fold and 2.35-fold, respectively, both of which were significantly higher than those observed in the TSB group. SoxR functions as a key regulator of the bacterial oxidative stress response, and its upregulation induces the expression of antioxidant enzymes, including KatG, thereby enhancing cellular defense mechanisms against oxidative damage [51]. The significant recovery of respiratory chain activity to 96.63% of the normal control level in the TSB-AHLs group (Figure 7) indicates that oxidative damage was effectively mitigated and intracellular redox homeostasis was largely re-established under this treatment condition.

**Figure 9:**
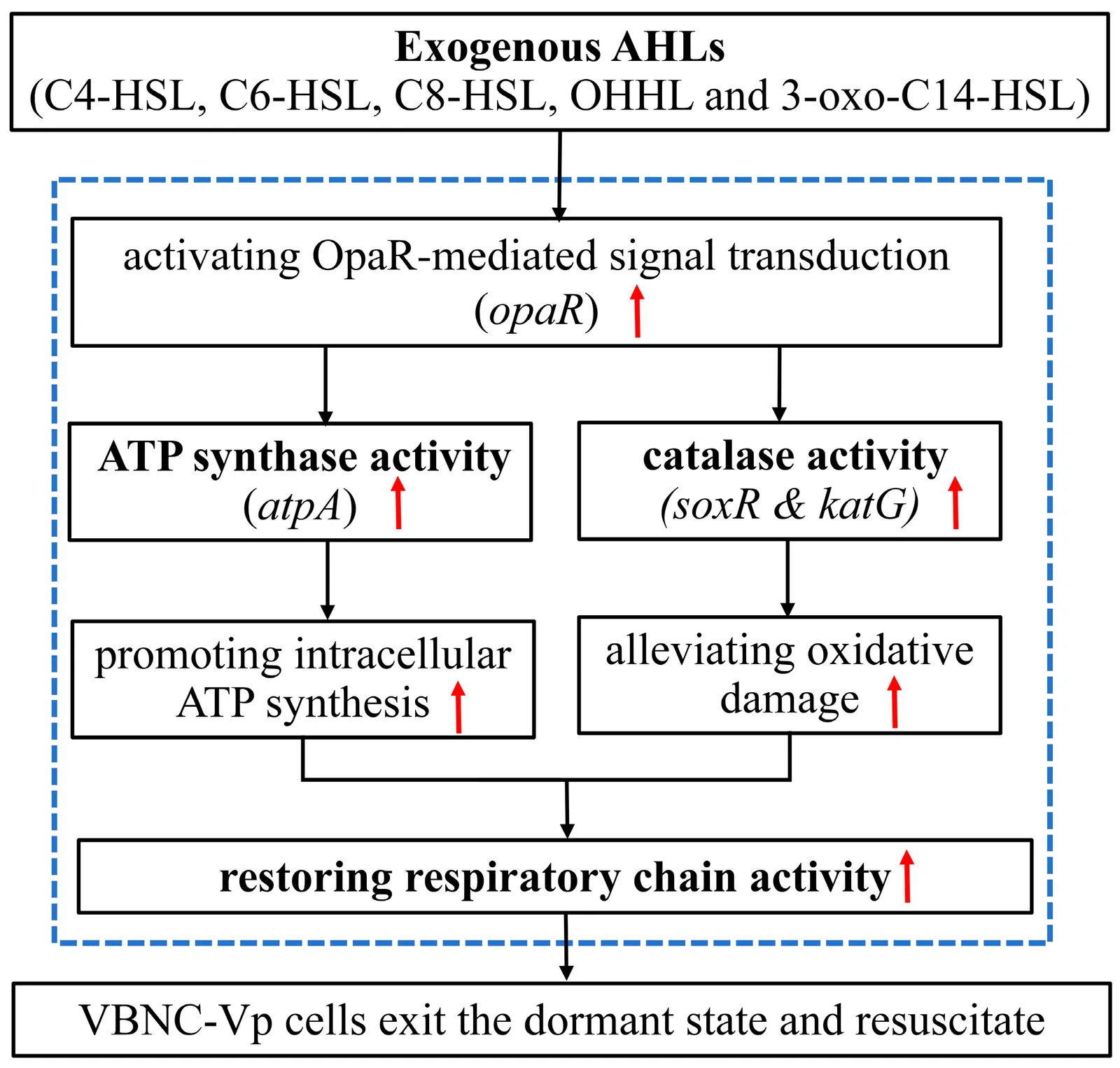
Proposed hypothetical model for the molecular mechanism by which exogenous AHLs may promote the resuscitation of VBNC *Vibrio parahaemolyticus*. Exogenous AHLs upregulate opaR expression and activate OpaR-mediated downstream signaling pathways. This activation initiates two synergistic pathways: the energy metabolism pathway (atpA ↑→ATP ↑) and the antioxidant defense pathway (soxR↑/katG↑→alleviation of oxidative damage). These two pathways act together to enhance respiratory chain activity, thereby facilitating the transition of VBNC cells from a dormant to an active state and enabling resuscitation. Red arrows indicate upregulation of gene expression; black arrows indicate sequential steps in the proposed pathway.

Based on these findings, a hypothetical molecular mechanism model for exogenous AHL-mediated resuscitation of VBNC-Vp is proposed (Figure 9). In this proposed model, exogenous AHLs (C4-HSL, C6-HSL, C8-HSL, OHHL and 3-oxo-C14-HSL) may be perceived by dormant cells and are proposed to promote OpaR-mediated signaling through the upregulation of opaR expression. This signaling pathway is hypothesized to involve two coordinated responses. The first response is associated with the upregulation of atpA, which coincides with increased intracellular ATP levels and may contribute to the energy supply required for cellular recovery during resuscitation. The second pathway involves the upregulation of soxR and katG, which is hypothesized to enhance antioxidant defense capacity, mitigates oxidative damage, and facilitates the restoration of respiratory chain function. According to this proposal, these two pathways act synergistically to promote the transition of VBNC Vp cells from a dormant to a metabolically active state, ultimately enabling effective resuscitation. It should be emphasized that the proposed model is hypothetical and is based on correlative evidence rather than direct mechanistic validation. Direct evidence supporting quorum sensing-mediated resuscitation, such as receptor-binding assays, gene knockout studies, or other functional analyses, is currently lacking and will be required to validate the proposed mechanism.

Notably, although the TSB-AHLs group exhibited strong resuscitation across all evaluated parameters, neither respiratory chain activity nor intracellular ATP levels fully reached those of the normal control group. This suggests that resuscitated cells may remain in a transitional phase of metabolic recovery rather than being fully equivalent to normal logarithmic-phase cells. This observation is consistent with the findings of Zhang et al. [52] in *E. coli*, where intracellular ATP levels in resuscitated VBNC cells remain lower than those of normal cells. Furthermore, whether resuscitated Vp regains pathogenicity remains to be determined. The principal virulence factors of Vp include thermostable direct hemolysin and thermostable direct hemolysin-related hemolysin, and the expression dynamics of the corresponding genes during the VBNC state and subsequent resuscitation warrant further investigation.

It should be noted that the classification of the VBNC state in this study is based on the conventional operational criteria of loss of culturability together with membrane integrity and basal metabolic activity. Although SYTO9/PI staining is widely used to distinguish viable from non-viable cells [31,32,33,34], it does not directly measure the ability of cells to resume growth. Therefore, the designation of the VBNC state in this study follows the standard operational criteria adopted in the field. The subsequent resuscitation experiments (Section 3.2) provide functional evidence supporting the recovery of culturability from this state, although the possibility of outgrowth from a very small residual culturable subpopulation cannot be completely excluded.

## 5. Conclusions

This study showed that, among the five resuscitation systems evaluated, TSB supplemented with a mixture of five AHLs was associated with the highest recovery efficiency of sodium hypochlorite-induced VBNC Vibrio parahaemolyticus. Exposure to 40 mg/L NaClO for 20 min effectively induced the VBNC state under the experimental conditions used in this study. Compared with sodium pyruvate, Tween 80, and heat treatments, the AHL-supplemented TSB medium was associated with greater recovery of culturability, ATP levels, and cellular morphology. In addition, RT-qPCR analysis revealed increased expression of opaR, atpA, soxR, and katG in the AHL-treated group. These findings suggest that exogenous AHL supplementation may facilitate the recovery of VBNC *V. parahaemolyticus* and that quorum sensing-related pathways, energy metabolism, and oxidative stress responses may contribute to this process. However, the present study demonstrates an association rather than a direct causal mechanism, and the specific signaling roles of individual AHLs remain to be elucidated.

These findings provide a theoretical basis for improving the detection and risk assessment of VBNC *V. parahaemolyticus* in seafood processing systems. Future studies should investigate the effects of individual AHLs and their combinations to identify the signaling molecules responsible for the observed responses; elucidate the molecular mechanisms underlying AHL-mediated recovery using genetic, biochemical, and integrated omics approaches; and further distinguish true resuscitation of VBNC cells from the outgrowth of a residual culturable subpopulation. In addition, validation of this strategy in complex seafood matrices under industrial processing conditions will help determine its practical applicability.

## Figures and Tables

**Table 1:** Quantitative morphometric analysis of Vp cell morphology under different treatment conditions.

Treatment Group	Cell Length (μm)	Cell Width (μm)	Aspect Ratio (Length/Width)
Normal control	1.68 ± 0.22	0.63 ± 0.08	2.68 ± 0.31
VBNC	0.82 ± 0.15 *	0.57 ± 0.06	1.45 ± 0.18 *
TSB	1.15 ± 0.17 *	0.60 ± 0.07	1.88 ± 0.20 *
TSB-HT	1.21 ± 0.16 *	0.61 ± 0.07	1.95 ± 0.18 *
TSB-SP	1.38 ± 0.18	0.62 ± 0.08	2.21 ± 0.19
TSB-T80	1.42 ± 0.20	0.63 ± 0.07	2.28 ± 0.21
TSB-AHLs	1.52 ± 0.19	0.62 ± 0.08	2.45 ± 0.22

Note: Values are presented as mean ± SD (n ≥ 50 cells per group). Asterisks indicate significant differences (*p* < 0.05) compared with the normal control group.

## Data Availability

The original contributions presented in the study are included in the article, further inquiries can be directed to the corresponding author.

## Supplementary Materials

The following supporting information can be downloaded at: https://www.mdpi.com/article/10.3390/microorganisms14081779/s1, Table S1. Composition and preparation conditions of the resuscitation treatment groups. Table S2. Primer sequences used for real-time quantitative PCR (RT-qPCR).
