# Supplementary material for: Effects of Nutritional Conditions and Exogenous N-Acyl-Homoserine Lactones on the Resuscitation of Sodium Hypochlorite-Induced Dormant Vibrio parahaemolyticus
Source: Microorganisms. 2026 Aug 12;14(8):1779. doi: 10.3390/microorganisms14081779 (PMC13515987; doi:10.3390/microorganisms14081779)
Supplement: Supplementary file 1 [file microorganisms-14-01779-s001.zip › microorganisms-4442335-supplementary.pdf]

Table S1. Composition and preparation conditions of the resuscitation treatment groups.

Groups	Basic medium	Supplement	Final concentration	Special treatment
TSB	TSB	—	—	—
TSB-heat treatment	TSB	—	—	heat shock at 72 °C for 10 s
TSB-sodium pyruvate	TSB	sodium pyruvate	2 mg/mL	—
TSB-Tween 80	TSB	Tween 80	5%（*v/v*）	—
TSB-signal molecule	TSB	C4-HSL, C6-HSL, C8-HSL, OHHL, and 3-oxo-C14-HSL	2 μmol/L	—

Note: TSB, tryptic soy broth. The TSB-heat treatment group was subjected to heat shock at 72 °C for 10 s after resuspension in TSB. Media without signaling molecules were sterilized by autoclaving at 121 °C for 15 min, whereas media containing sodium pyruvate, Tween 80, or AHLs were sterilized by filtration through 0.22 μm membrane filters. All media were stored at 4 °C before use. Each treatment was performed in triplicate.

Table S2. Primer sequences used for real-time quantitative PCR (RT-qPCR).

Gene	Primer type	Sequence (5′ to 3′)	Product length (bp)
opaR	Forward	ATCAGCAGCGGAAACAAGGT	152
	Reverse	TTGCTCCGATAGCGTTGACC	
atpA	Forward	ACACTGACCTTTGCCGATGA	168
	Reverse	GGTGAACGGTGGAGTTGAGT	
soxR	Forward	TGAACGAGTGGCAGATAGCC	145
	Reverse	CCGATTCGCTAACACCAGGT	
katG	Forward	TGGCGAGTTTGAGCCAGATA	156
	Reverse	TTGGTGAAGCCGTACGTGAT	
16S rRNA	Forward	GCTAAGCCCTTGACAGGCTA	185
	Reverse	CCATGATGTGACGGGCGGTG	

Note: The target genes included the quorum sensing regulator (*opaR*), ATP synthase α-subunit (*atpA*), oxidative stress regulator (*soxR*), catalase-encoding gene (*katG*), and the reference gene (*16S rRNA*). Primers were designed based on the *Vibrio parahaemolyticus* genome sequence and synthesized by Sangon Biotech Co., Ltd. (Shanghai, China). All reactions were performed in triplicate, and relative expression levels were calculated using the $2^{-\Delta\Delta Ct}$ method with 16S rRNA as the internal reference gene.
